# Effects of Five Years of Treatment of Onchocerciasis with Ivermectin under Community Guidelines in Resurgent Areas of Burkina Faso: A before-and-after Analysis

**DOI:** 10.3390/tropicalmed9090207

**Published:** 2024-09-09

**Authors:** Micheline O. Ouedraogo, Ivlabèhirè Bertrand Meda, Karifa Kourouma, Fanny Yago Wienne, Dieudonné Nare, Clarisse Bougouma, Justin Compaore, Seni Kouanda

**Affiliations:** 1 Helen Keller International, Ouagadougou 06 P.O. Box 9515, Burkina Faso; fyago-wienne@hki.org (F.Y.W.); dnare@hki.org (D.N.); 2 African Institute of Public Health (IASP), Ouagadougou 12 P.O. Box 199, Burkina Faso; medabert@yahoo.fr (I.B.M.); senikouanda@gmail.com (S.K.); 3 National Center for Training and Research in Rural Health of Maferinyah (CNFRSR), Maferinyah P.O. Box 2649, Guinea; kkourouma@maferinyah.org; 4 National Program for the Control of Neglected Tropical Diseases, Ouagadougou 06 P.O. Box 9515, Burkina Faso; obclarisse@hotmail.com (C.B.); justincompaore@yahoo.com (J.C.)

**Keywords:** onchocerciasis, ivermectin, resurgent areas, microfilarodermia, community microfilarial load, standardized prevalence, community guidelines, Burkina Faso

## Abstract

Background: Almost the entire country of Burkina Faso was endemic to onchocerciasis. Onchocerciasis control efforts thus brought the prevalence of *O. volvulus* to a level where the disease was no longer a public health problem in 2002. A resurgence of onchocerciasis cases has been observed in two regions (Cascades and the Southwest) located around several river basins in 2010–2011. In accordance with WHO guidelines for the management of resurgent cases, community-directed treatment with ivermectin (CDTI) was implemented in the affected areas. The aim of this study was to determine the effects of this intervention on parasitological indices of onchocerciasis, depending on the distance between villages and rivers. Methodology: We conducted a paired pre-post study using aggregated village-level data from two cross-sectional surveys conducted in each region. A Wilcoxon signed-rank test was used to compare the standardized microfilarodermia prevalence and community microfilarial load (CMFL). Results: A total of 43 villages in 6 health districts, in the Southwest (18) and Cascades (25) regions were included in the study. The key findings were that standardized microfilaria prevalence and CMFL decreased significantly after the implementation of CDTI in both regions (*p* < 0.0001). The median standardized microfilaria prevalence was 2.8 [interquartile range (IQR): 0.2–6.6] before CDTI and 0.72 [IQR: 0.0–2.17] after CDTI. The results showed also a decline in standardized microfilaria prevalence and CMFL in all villages, regardless of the distance separating the village from the streams. However, the results were not statistically significant for the villages located 5 km or more from streams (*p* = 0.0816 and 0.0542 for standardized microfilaria prevalence and CMFL, respectively). Conclusion: Our results thus show that the implementation of effective CDTI could stop the transmission of *O. volvulus* in these two regions. The main challenge for stopping transmission could be the migration of populations to neighboring countries and migration of the vector from one country to another, as Burkina Faso shares some river basins with neighboring countries.

## 1. Introduction

Onchocerciasis, a parasitic infection caused by the nematode *Onchocerca volvulus* and transmitted by the small biting fly *Simulium damnosum s.l*, is a major public health problem due to the irreversible complications (blindness, debilitating itching) that result [1]. It is one of the 20 neglected tropical diseases (NTDs) to be eliminated by 2030 [1,2]. According to the World Health Organization (WHO) [3], the vast majority of the 26 million people infected with onchocerciasis worldwide (99%) live in sub-Saharan Africa [3,4].

The advent of vector control programs implemented since 1974 in West Africa, and later associated with mass treatment campaigns with ivermectin implemented in the 1990s, have improved the socio-economic conditions of populations through the elimination of the disease as a public health problem in some countries [4,5]. This ivermectin-based treatment, initially provided by mobile teams, is now led by the community in an approach called community-directed treatment (CDTI) [4].

However, some experts believe that it will not be possible to eliminate onchocerciasis in Africa with mass treatment with ivermectin alone because of the more complex epidemiology in region compared with Latin America [6]. Indeed, the outbreaks in Latin America were localized with low to moderate endemicity and the vectors were less effective in transmitting infection compared to those in Africa [6]. This could explain why the results of several years of CDTI appear more effective in sub-Saharan Africa. For example, studies in Niger, Senegal, Nigeria, and Ethiopia have shown that at least 15 years of mass treatment with ivermectin were required to interrupt *O. volvulus* transmission and achieve prevalence rates of zero [7,8,9]. Similarly, other studies, in Cameroon and Togo, showed that more than 15 years of CDTI failed to interrupt onchocerciasis transmission [10,11,12,13]. Several hypotheses have been put forward, including insufficient coverage of the population eligible for CDTI and maintenance of the infectious life cycle through human or vector migration.

Almost the entire country of Burkina Faso was endemic to onchocerciasis, with a prevalence of 60–80%, except for the extreme north [14]. Onchocerciasis control was mainly based, as elsewhere in West Africa, on vector control using larvicides from 1974 until the 1990s. Ivermectin treatment was used in the Bougouriba basin in 1996 and in the Nakambé and Sissili basins from 1992 to 1998 with the aim of controlling resurgence [4,14]. Onchocerciasis control efforts thus brought the prevalence of *O. volvulus* to a level where the disease was no longer a public health problem, and a national onchocerciasis control program was set up in 2002 to maintain the gains made. In addition, starting in 2000, Burkina Faso implemented mass distribution of ivermectin to control lymphatic filariasis which was scaled up starting in 2004 [14], covering all health districts in the country.

A resurgence of onchocerciasis cases has been observed in two regions (Cascades and the Southwest) located around several river basins in the 2010s [14,15,16]. In accordance with WHO guidelines for the management of resurgent cases, CDTI was implemented in the affected areas of Cascades and Southwest regions [15]. Five years after the start of CDTI in these regions, epidemiological surveys were conducted to assess progress in controlling the resurgence of the disease. Taking advantage of the pre-CDTI survey and the last epidemiological survey, the objective of this study was to evaluate the effects of five years of CDTI on the parasitological indices of onchocerciasis. The study is unique in that it concerns CDTI conducted in the context of resurgence control, unlike other studies conducted in other sub-Saharan African countries that were based on initial disease control interventions [8,17,18]. Furthermore, this resurgence occurred despite the use of ivermectin for lymphatic filariasis control and therefore the efficacy of CDTI in this context is questionable.

## 2. Methods

### 2.1. Study Design

This was a paired pre-post intervention study without control groups in the Cascades region and the Southwest region of Burkina Faso.

### 2.2. Study Setting

Burkina Faso, a country located in West Africa, borders Côte d’Ivoire, Mali, Ghana, Togo, Benin, and Niger. It had a population of 20,487,979 in 2019, of which more than three-quarters live in rural areas and 39.3% below the poverty line [19]. In addition, 56.2% of the population are farmers and/or skilled workers in agriculture, forestry, or fishing [20].

Climatically, Burkina Faso is divided into 3 zones: a Sahelian zone which is located in the north of the country, a Sudanese-Sahelian zone running from the east to the north and including the center of the country, and a Sudanese zone located in the southwest [21]. The vegetation is much more abundant in the Sudanese zone of the country where the two onchocerciasis endemic regions are located.

Onchocerciasis endemic regions are crossed by some of these tributaries and basins. In the Southwest region, the districts where onchocerciasis are endemic are crossed by the Mouhoun (Bas Mouhoun) and Bougouriba rivers. As for the Cascades region, the 2 endemic districts are crossed by the Comoe river.

Only the Southwest and Cascades regions, out of the 13 regions of the country, are endemic to onchocerciasis, with districts having an endemicity threshold above 5% [22]. The endemic districts are those of Gaoua, Batié, Diébougou, and Dano (Southwest region) and Banfora and Mangodara (Cascades region).

### 2.3. Implementation of the CDTI by the National Program for the Fight against NTDs in Burkina Faso

**Figure 1:**
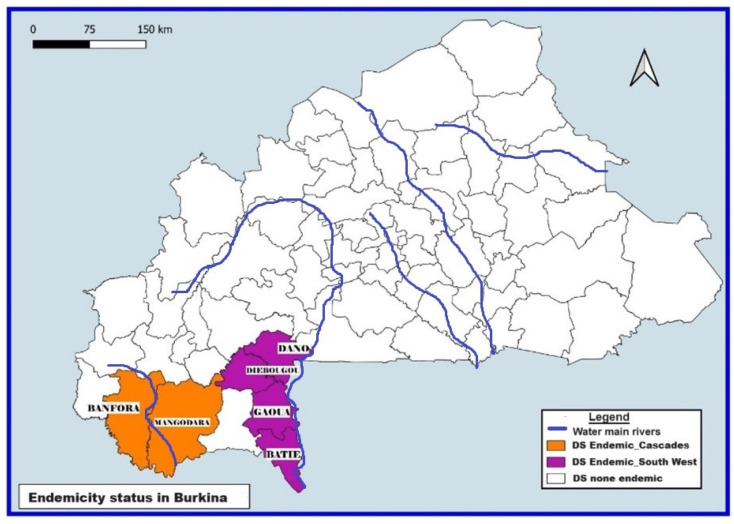
Regions covered by CDTI in Burkina Faso.

Implementation of the CDTI was resumed in 2011 and 2013 in the Cascades and Southwest regions, respectively; after that, the 2010 (Cascades) and 2011 (Southwest) epidemiological assessments showed a high prevalence of the disease (see Figure 1). CDTI is implemented biannually in both regions except in the years when an epidemiological survey is conducted (2016 for the Cascades and 2018 for the Southwest). For the study periods, 10 and 9 rounds of CDTI were conducted, respectively, in the endemic districts of the Cascades and the Southwest region by the Neglected Tropical Diseases Program (NTDP) of the Ministry of Health.

The CDTI is a strategy implemented by communities and evaluated by community monitors. During CDTI campaigns, community-based distributors are responsible for administering treatment by name in all onchocerciasis-endemic villages. Experience has shown that this method works best if the communities themselves choose how they want to deliver the treatment and who will be responsible for it. Prior to the implementation of each CDTI campaign, the actors are trained in a cascade from the regional level to the community distributors (CDs). The administration of ivermectin is carried out by the CDs under the supervision of those responsible at the health facilities, the districts, and the regional teams. Social mobilization is carried out at the regional, health district, and community levels in order to encourage greater support from the population.

### 2.4. Data Sources: Epidemiological Surveys Implemented in the Framework of Onchocerciasis Surveillance

The data come from four epidemiological surveys (2 per region) [23,24,25,26]. An epidemiological survey is a descriptive cross-sectional survey that aims to determine the prevalence of onchocerciasis in communities. The pre-intervention surveys were carried out between January and April 2010 and between January and February 2011 in 28 villages in the Cascades and 20 villages in the Southwest, respectively. The samples were made up of sentinel villages and non-sentinel villages, but the reports do not specify how the latter were selected. A sentinel village is village that is periodically assessed to check the prevalence of onchocerciasis. The post-intervention surveys were conducted between September and October 2016 and in November 2018 in the Cascades and Southwest regions, respectively. The post-surveys covered 28 villages in the Cascades, 25 of which had already been surveyed in 2010, and 29 villages in the Southwest, 18 of which had already been surveyed in 2011.

In each village, the population was censused and all those eligible and consenting were surveyed. Thus, 5044 persons out of 9050 identified and 6301 out of 11,647 were surveyed in the Cascades region before and after the intervention, respectively. In the Southwest, 3760 individuals were surveyed pre-intervention and 5606 out of 8522 were surveyed post-intervention.

In the pre-intervention surveys, the method used for the assessment of onchocerciasis prevalence was just the examination of exsanguinated skin biopsies (a skin flap covering the superficial layers of the dermis). But, in post-intervention surveys, the parasite antigens using the rapid tests, *Onchocerca volvulus* OV-16 antigen–antibody test (OV-16) was also used. The use of rapid tests in epidemiological investigations is a component of the new WHO guidelines for the elimination of onchocerciasis as a public health problem [27].

The method for exsanguinated skin biopsy examination is as follows. A 1–2 mg skin flap is taken with Holtz forceps, including the superficial layers of the dermis, and is placed in distilled or physiological water. The emitted microfilariae are observed fresh under the microscope or with a binocular microscope. The reading of the biopsies with a magnifying glass is performed 30 min after incubation in distilled water. The populations from whom skin biopsies were taken were those aged 5 years and over.

OV16 tests are performed in children aged 2 to 9 years. These tests are based on the detection of IgG4 antibodies reacting against the OV16 parasite antigen in a drop of blood taken from a fingertip. The OV16 tests are read after 20 min and again after 24 h.

### 2.5. Study Population

Inhabitants (aged ≥5 years) residing in endemic villages in the health districts of Banfora and Mangodara in the Cascades region and Batié, Gaoua, Dano, and Diébougou in the Southwest region constituted our study population. Thus, a total of 43 villages were involved in the study. The villages that were selected were those that participated in the epidemiological surveys before the implementation of CDTI (in 2010 and 2011) and after five years of treatment.

### 2.6. Study Variables

Two outcome variables are considered in this study: the standardized microfilarodermia prevalence rate and the community microfilarial load. The crude microfilarodermia prevalence is the percentage of microfilaria carriers in a sample [28]. Standardized prevalence allows the crude microfilarodermia prevalence to be corrected by applying it to the standard population to take account of variability due to sex and age. The community microfilarial load (CMFL) is the sum of the microfilarial loads of all the individuals in a sample divided by the number of individuals in the sample. This indicator is calculated only for adults aged 20 and over. This is a much more sensitive indicator than prevalence. Both indicators were available per village. The *Onchocerca volvulus* OV-16 antigen–antibody test (OV-16) was not included as an outcome variable because there was no pre-intervention measurement.

Exposure was the intervention taking the value 0 for the period before and 1 for the period after the implementation of CDTI. The distance between the village and the river (coded 1 if the distance < 5 km and 0 if ≥5 km) was treated as an effect modifier variable. We hypothesized that the villages located less than 5 km from watercourses are those where the endemicity of onchocerciasis may be greater due to the permanent presence of black flies.

### 2.7. Data Analysis

Data in Excel format (version 2019) were processed and then imported into Stata V.15.2 software (Stata Corporation, College Station, TX, USA) for analysis. We first plotted the observed trajectories of all villages. We then used boxplots to examine the observed pre- and post-CDTI prevalence and community microfilarial load (CMFLs) for the entire sample, by region, and according to the distance of a village from the streams. To assess the effects of CDTI on the standardized microfilarodermia prevalence and the community microfilaria burden, we used the Wilcoxon signed-rank test as the distribution of the two outcome variables are asymmetric. We considered the effects to be different across regions and village distances to rivers and we presented the results unstratified and stratified by region and then by the distance of the villages from the rivers.

### 2.8. Ethical Considerations

This study was a secondary analysis of data from the NTDP’s CDTI implementation activities. The data used were anonymized and did not include information that would allow for the identification of respondents. In addition, for all studies related to the elimination of onchocerciasis, the PNMTN had approval from the National Health Research Ethics Committee, signed in 2009. This approval has also been used for the publication of other studies [29]. However, prior to the implementation of the surveys, the teams sensitized the populations on onchocerciasis and its consequences and on the need to monitor its endemicity. Following this, the participants in the survey gave oral or written consent prior to sampling. The database was kept on the principal investigator’s password-protected computer.

## 3. Results

### 3.1. Description of the Sample

The 43 villages were distributed across six health districts (Batie, Dano, Diebougou, Gaoua, Banfora, and Mangodara). More than half (25/43) of the villages were located in the districts of Banfora and Mangodara in the Cascades region. The Banfora district alone accounted for 20 of the 43 villages surveyed (Table 1).

### 3.2. Standardized Microfilarodermia Prevalence before and after the CDTI

**Figure 2:**
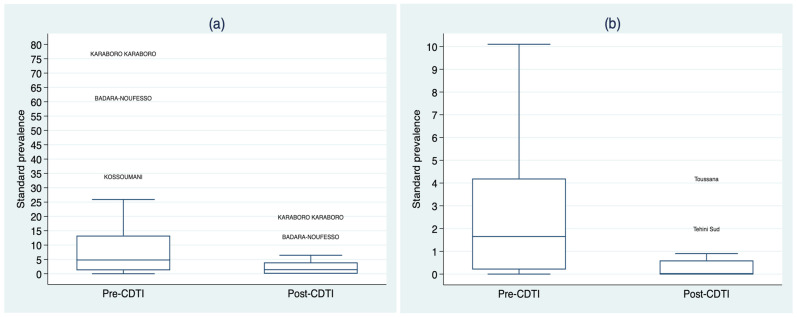
Distribution of standardized microfilarodermia prevalences between pre- and post-CDTI by region: (**a**) Cascades and (**b**) Southwest.

Standardized microfilarodermia prevalences were skewed to the right, both in the Cascades and the Southwest region and both pre- and post-CDTI. Standardized microfilarodermia prevalences were higher in the Cascades region than in the Southwest, both before and after the intervention (Figure 2 and Table 2). Standardized microfilarodermia prevalences were also higher in villages located less than 5 km from watercourses than in those located 5 km or more. Figure 2 also shows greater variability in standardized microfilarodermia prevalences in the villages of Cascades region compared with the Southwest. A similar variability is observed when comparing villages located less than 5 km from watercourses with those located 5 km or more away (See Supplementary Figure S1).

### 3.3. Distribution of Community Micofilarial Load (CMFL) before and after CDTI

**Figure 3:**
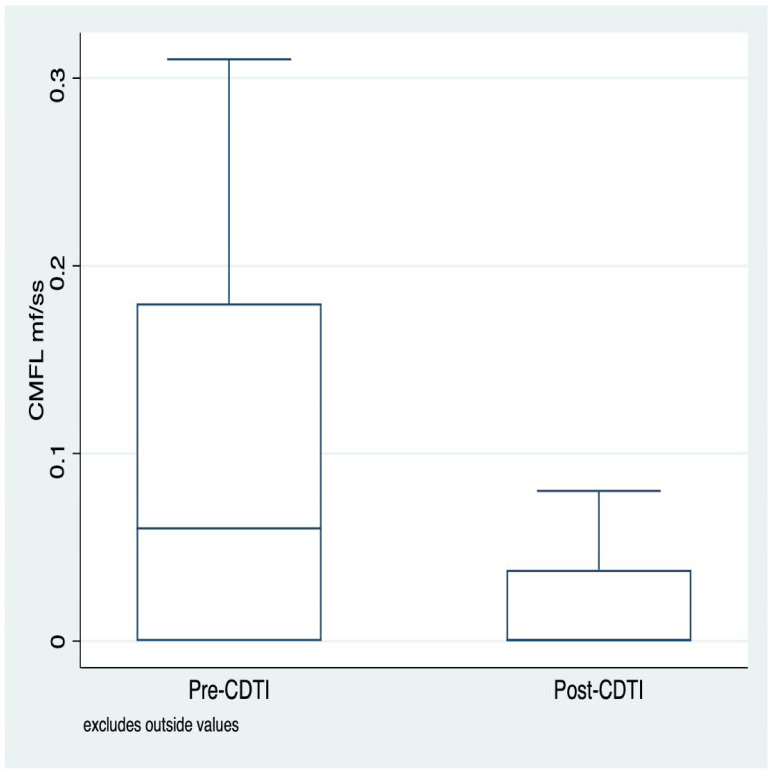
Distribution of pre- and post-CDTI microfilarial loads for villages in the Cascades and Southwest regions, Burkina Faso.

Similar to the standardized microfilarodermia prevalence rates, the distribution of the community microfilarial load (CMFL) was positively skewed (Figure 3).

### 3.4. The Effects of CDTI on Standardized Microfilaria Prevalence and CMFL

**Figure 4:**
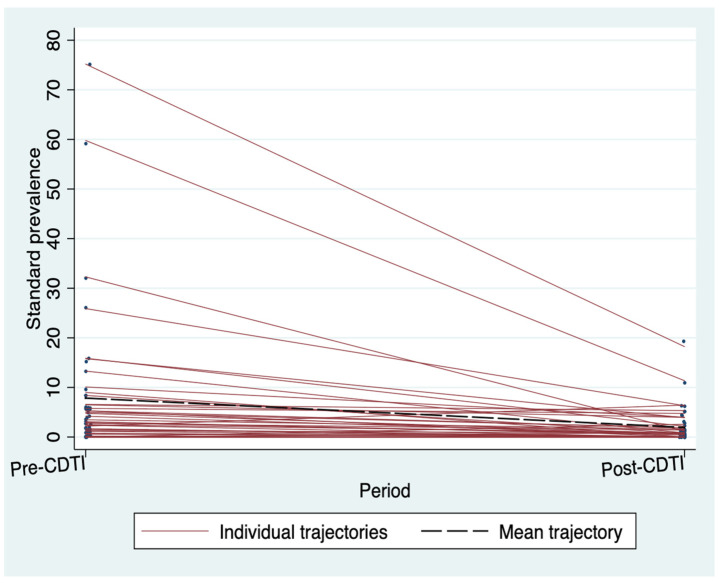
Standardized microfilarodermia prevalence trajectories of the 43 villages that benefited from CDTI in the two regions, Burkina Faso.

Figure 4 shows that the villages with the highest pre-CDTI standardized microfilarodermia prevalences are those that have seen the greatest reductions. The evaluation of the effects of CDTI showed a statistically significant reduction in standardized prevalence and CMFL after the five years of CDTI implementation in both regions. This significant reduction in parasitological indices was observed in the villages located less than 5 km from the river. Villages located 5 km or more from watercourses also experienced a reduction in parasitological indices, but the results were not statistically significant (Table 2).

## 4. Discussion

This study was conducted to analyze the effects of CDTI implementation on changes in the prevalence of *O. Volvulus* in two regions in Burkina Faso that had seen a recrudescence.

The key findings were that standardized microfilarodermia prevalence, skin biopsy prevalence, and CMFL decreased significantly after the implementation of CDTI in both regions. There was no significant difference between the decline in parasitological data within the two regions. A greater decline in the trajectory of prevalence was observed for villages located less than 5 km from the watercourse compared to villages located more than 5 km from the watercourse.

We conducted an analysis to determine the effects of CDTI on parasitological indices of onchocerciasis in these two areas and we also analyzed the data in relation to the location of the village in relation to the river to see if this was a factor in the decrease in prevalence. This in-depth analysis allowed us to obtain satisfactory results despite the small size of our sample as one of the limitations of our study was the small sample. We were unable to obtain individual data from the epidemiological studies and had to work on the basis of villages as our unit of measure. In addition, we had to assign district therapeutic coverage rates to villages due to the lack of coverage data at the village level.

Our results showed a decline in standardized microfilarodermia prevalence and CMFL in all villages involved in the study. This was shown by the trajectories and the distribution by villages before the implementation of CDTI and after five years of treatment. The reduction in standard microfilarodermia prevalence and CMFL could be attributed to the effects of CDTI. For example, for standardized prevalence, the reduction was nearly −6 with a 95% confidence interval of [−9.6 to −2.2]. However, we did not carry out multivariate regression to incorporate confounding variables. Confounding variables such as the environment of the region, the density of the black fly population, the behavior of the communities, and many others could influence the results. Nonetheless, previous research has found that ivermectin is an effective treatment for onchocerciasis microfilariae to stop the transmission of onchocerciasis. This discovery allowed vector control for the elimination of onchocerciasis to be suspended in order to strengthen efforts to implement ivermectin treatment [30]. The findings also support the view that mass treatment with ivermectin is better in several respects compared to vector control. It has an impact on morbidity and it helps to reduce disease transmission [31]. The significant decline in prevalence after five years of treatment in the two regions of Burkina Faso suggests that the implementation of CDTI could lead to a cessation of onchocerciasis transmission within the 15-year time frame recommended in the studies. Indeed, according to mathematical models, in the event of a resurgence, CDTI conducted for 15 years with a coverage rate of at least 65% would be sufficient to stop the recrudescence and spread of onchocerciasis [4]. The fact that the standardized prevalences of microfilarodermia were higher before the implementation of CDTI in villages located less than 5 km from watercourses than in those located 5 km or more away is consistent with the fact that the density of the Simulium population is higher, resulting in a higher number of infesting bites. This implies that prevalence monitoring activities are most often carried out in these villages.

Although the decline in standardized microfilarodermia prevalence rates and CMFL appeared to be greater in the Cascades region than in the Southwest region, this was not statistically significant. This could be due to the small sample size which did not allow for statistical power. We have no factual data to explain the prevalence rates. However, the high prevalence of onchocerciasis in the Cascade region before the resumption of CDTI could explain the significant reduction in CMFL. Indeed, CDTI is always associated with a reduction in prevalence and CMFL even when baseline prevalences are high. Studies have shown area differences in the time taken to total elimination of onchocerciasis but these were usually conducted at the country level with large samples. For example, in Abu Hamed, Sudan, onchocerciasis was declared eliminated after 14 years of annual and then biannual treatment with ivermectin [31]. In Geba Valley, Guinea-Bissau, it took just six years of treatment [4]. In our study, we dealt with two regions of the same country that were located in the same geographical area. These two regions have the same type of climate and vegetation with people who have the same habits in terms of professional activities (mainly agriculture, livestock, and fishing), culture, and migration. This could also explain the lack of significant difference in the decline in prevalence. In addition, the implementation of CDTI in both regions was coordinated by the same team at the national level. The strategies used to achieve adherence and treatment goals were probably the same in both regions, as evidenced by the satisfactory treatment coverage.

We also did not observe a significant difference in the decrease in standardized prevalence and CMFL of villages located less than 5 km from the rivers and those located more than 5 km away. However, we know that the frequency of dermal microfilariae is a function of the number of adult filariae and hence the number of infective larvae transmitted to the subject by the bites of infective female black flies [32]. The proximity of villages to watercourses leads to greater simulidean nuisance and hence higher transmission.

The small size of our sample was a limitation of our study. It did not allow us to demonstrate the effect of proximity to waterways on the reduction in the prevalence of onchocerciasis, despite extensive analysis. Another limitation was the absence of data on the therapeutic coverage of the villages concerned by the study, which obliged us to attribute the therapeutic coverage of the districts to the villages. In addition, we did not have sufficient data on the effect of population migrations on the prevalence of onchocerciasis, even though it has been observed that which are.

However, the results obtained show that the implementation of CDTI in the two regions has produced satisfactory results, and the reduction in the endemicity of onchocerciasis will certainly lead to other programmatic decisions such as the implementation of pre-stop and full-stop surveys in these two regions.

## 5. Conclusions

Our study showed the effects of CDTI on the decrease in standardized microfilarodermia prevalence and CMFL in all the villages concerned. CDTI was implemented for at least five years between the first and second epidemiological assessments, with satisfactory treatment coverage in all districts. Our results thus show that the implementation of effective CDTI could stop the transmission of *O. volvulus* in these two regions. However, CDTI is continuing in both regions, and in accordance with recent WHO guidelines on the elimination of onchocerciasis, a pre-stop epidemiological survey, followed by full-stop surveys (epidemiological and entomological), will be carried out before treatment is stopped in these regions. The main challenge for stopping transmission could be the migration of populations to neighboring countries. It was observed that most of the people who tested positive during the epidemiological surveys after the five years of treatment had stayed outside Burkina Faso for a longer or shorter period of time. Effective strategies should be put in place to track these individuals as soon as they return to the country in order to prevent them from transmitting onchocerciasis to populations that are regularly treated. In addition to population migration, the migration of the vector from one country to another (as Burkina Faso shares some river basins with neighboring countries) may also delay the cessation of transmission. Future studies are needed to assess the impact of these migration patterns on preventing *O. Volvulus* transmission.

## Figures and Tables

**Table 1:** Number of selected villages per health district.

Region	Health District	Number of OV Endemic Villages	Number of Villages Selected for Survey
Cascades	Banfora	103	20
	Mangodara	20	5
Southwest	Batié	111	8
	Dano	108	3
	Diébougou	47	5
	Gaoua	11	2

**Table 2:** Comparison of standardized microfilarodermia prevalence and community microfilarial load (median and interquartile range) by region and distance of village from river between pre-CDTI and post-CDTI periods, Burkina Faso.

	Pre-CDTI Period	Post-CDTI Period	*p*-Value *
Cascades and Southwest (N. of villages)	43	43	
Standardized microfilarodermia prevalence	2.8 (0.2–6.6)	0.72 (0.0–2.17)	<0.0001
Community microfilarial load	0.06 (0.0–0.18)	0.0005 (0.0–0.038)	<0.0001
Cascades (N. of villages)	25	25	
Standardized microfilarodermia prevalence	4.8 (1.2–13.3)	1.41 (0.0–3.96)	0.0008
Community microfilarial load	0.08 (0.0–0.23)	0.021 (0.0–0.054)	0.0001
Southwest (N. of villages)	18	18	
Standardized microfilarodermia prevalence	1.65 (0.2–4.2)	0.0 (0.0–0.6)	0.0011
Community microfilarial load	0.04 (0.0–0.14)	0.0 (0.0–0.01)	0.0009
Less than 5 km from river (N. of villages)	32	32	
Standardized microfilarodermia prevalence	3.85 (1.0–9.55)	0.77 (0.0–3.035)	<0.0001
Community microfilarial load	0.075 (0.015–0.235)	0.005 (0.0–0.044)	<0.0001
5 km and more from river (N. of villages)	11	11	
Standardized microfilarodermia prevalence	2.5 (0.0–3.1)	0.6 (0.0–1.41)	0.0816
Community microfilarial load	0.04 (0.0–0.08)	0.0005 (0.0–0.028)	0.0542

* Wilcoxon signed-rank test; CDTI = community directed treatment by ivermectin.

## Data Availability

The data used in this study belong to the NTDP in Burkina Faso. They are available upon reasonable request.

## Supplementary Materials

The following supporting information can be downloaded at: https://www.mdpi.com/article/10.3390/tropicalmed9090207/s1, Figure S1: Distribution of standardized microfilarodermia prevalences between pre- and post-CDTI according to the distance of the villages from the watercourses: (a) Less than 5 kms and (b) 5 kms and more.
