# Supplementary material for: Effects of Five Years of Treatment of Onchocerciasis with Ivermectin under Community Guidelines in Resurgent Areas of Burkina Faso: A before-and-after Analysis
Source: Trop Med Infect Dis. 2024 Sep 9;9(9):207. doi: 10.3390/tropicalmed9090207 (PMC11435634; doi:10.3390/tropicalmed9090207)
Supplement: Supplementary file 1 [file tropicalmed-09-00207-s001.zip › tropicalmed-3172893-supplementary.pdf]

Supplementary material

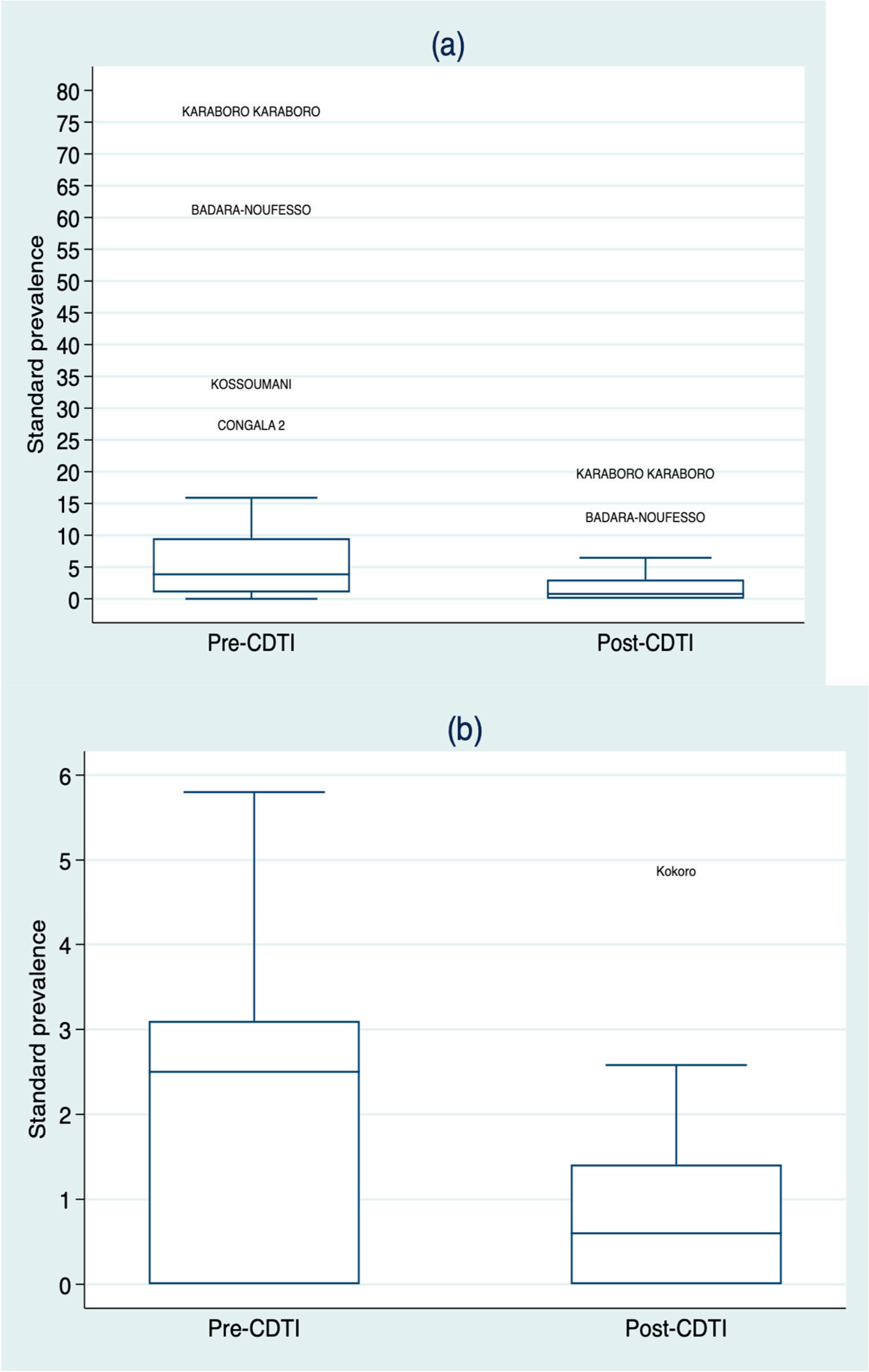

Figure S1. Distribution of standardized microfilarodermia prevalences between pre- and post-CDTI according to the distance of the villages from the watercourses: (**a**) Less than 5 kms and (**b**) 5 kms and more.
